# Mathematical modelling of the action potential of human embryonic stem cell derived cardiomyocytes

**DOI:** 10.1186/1475-925X-11-61

**Published:** 2012-08-28

**Authors:** Michelangelo Paci, Laura Sartiani, Martina Del Lungo, Marisa Jaconi, Alessandro Mugelli, Elisabetta Cerbai, Stefano Severi

**Affiliations:** 1 Biomedical Engineering Laboratory – D.E.I.S, University of Bologna, Via Venezia 52, Cesena, 47521, Italy; 2 Center of Molecular Medicine (C.I.M.M.B.A.), University of Firenze, Viale Pieraccini 6, Florence, 50139, Italy; 3 Department of Pathology and Immunology, University of Geneva, Rue Michel-Servet 1, Geneva 4, 1211, Switzerland

**Keywords:** Embryonic stem cells, Computer simulation, Action potential, Pharmacology

## Abstract

**Background:**

Human embryonic stem cell derived cardiomyocytes (hESC-CMs) hold high potential for basic and applied cardiovascular research. The development of a reliable simulation platform able to mimic the functional properties of hESC-CMs would be of considerable value to perform preliminary test complementing in vitro experimentations.

**Methods:**

We developed the first computational model of hESC-CM action potential by integrating our original electrophysiological recordings of transient-outward, funny, and sodium-calcium exchanger currents and data derived from literature on sodium, calcium and potassium currents in hESC-CMs.

**Results:**

The model is able to reproduce basal electrophysiological properties of hESC-CMs at 15 40 days of differentiation (Early stage). Moreover, the model reproduces the modifications occurring through the transition from Early to Late developmental stage (50-110, days of differentiation). After simulated blockade of ionic channels and pumps of the sarcoplasmic reticulum, Ca^2+^ transient amplitude was decreased by 12% and 33% in Early and Late stage, respectively, suggesting a growing contribution of a functional reticulum during maturation. Finally, as a proof of concept, we tested the effects induced by prototypical channel blockers, namely E4031 and nickel, and their qualitative reproduction by the model.

**Conclusions:**

This study provides a novel modelling tool that may serve useful to investigate physiological properties of hESC-CMs.

## Background

Human Embryonic Stem Cells (hESCs) are pluripotent cells derived from the blastocyst stadium of human embryos, having the potential to differentiate in all the three embryonic germ layers [1]. Many studies have been carried out to identify the most advantageous strategies to drive the differentiation towards the desired cell phenotypes thus allowing valuable investigations in basic research and suggesting useful perspectives for regenerative purposes. In the cardiovascular field, hESCs provide a powerful tool to clarify key developmental steps of cardiac embryogenesis [2], to develop reliable in vitro models for drug toxicity screening [3] and are considered a promising source for cell-based therapies in pathologies such as myocardial infarction or pace-maker center dysfunction [4]. Studies involving hESCs differentiated toward the cardiac phenotype are rather demanding due to difficulties such as i) low efficiency process of differentiation [5]; ii) dishomogeneity of cell phenotypes; iii) laborious phenotypic characterization, e.g. via patch-clamp or multicellular and multi-electrode array recordings [6-8]. Another complication arises from the observation that hESC-derived cardiomyocytes (hESC-CMs), like fetal cardiomyocytes (CMs), are electrophysiologically immature [6]; their properties evolve during in vitro culturing [6], a phenomenon which appears to be regulated by interactions with non-cardiomyocytes in embryoid bodies (EBs) [9].

To date extensive information is available mostly as unsystematic mass of basic electrophysiological properties of different hESC lines differentiated toward the cardiac lineage and on the modifications occurring during maturation or upon exposure to different drugs or chemicals. Nonetheless, no attempt was done to systematize current knowledge to fully evaluate the impact of individual key ionic currents and of excitation-contraction coupling mechanisms on basic physiology in hESC-CMs [10-13].

Computational modelling represents a consolidated approach in cardiac research to simulate the electrophysiology of single cell or cell-made tissue [14] and the modifications induced by chemicals and drugs. This approach usually complements in vitro and in vivo experimentation to create a compelling tool able to predict physiological responses, abnormal reactions to drug application and to formulate new hypotheses.

The aim of this work is to develop a computational model of action potential (AP) of H1-hESC-CMs allowing to follow the maturation process. Due to the shortage of measurements, especially at advanced developmental stages, this model can help to infer developmental mechanisms not obvious from the bare measurements.

Data on membrane ionic currents for this cell line coming from our original measurements of transient outward potassium, funny, sodium-calcium exchanger currents, and data from literature on sodium, calcium and potassium currents in hESC-CMs were integrated into the model. To take into account the presence of non-cardiac cells in intact EBs, a further model assessment is proposed by coupling the hESC-CM model with modelled fibroblasts and evaluating their impact on the AP. The formulated model simulates i) the main basic AP features and ii) the developmental changes documented during in vitro maturation.

## Methods

Methods for hESC-CM culture, differentiation and electrophysiological recording are described in Additional file 1. hESC-CM were included in the Early or Late group according to differentiation time, i.e. from 15-40 and 50-110-days, respectively [6].

### Model of *hESC-CM AP* and its transition from *Early to Late* stage of development

The starting point in developing the hESC-CM model was a modified version [15] of the TenTusscher model of human adult ventricular CM [16], this parent model was then largely modified by changing the formulation of almost all the currents to incorporate all the available data on hESC-CMs and by adding two currents (I_f_ and I_CaT_) that are not present in the adult ventricle. Following the classical Hodgkin-Huxley formulation [17], the cell electrophysiological behaviour is described by Eq. 1:

(1) $$\frac{dV}{dt}=-{\frac{I_{\mathrm{ion}}}{C_{m}}}_{\text{,}}$$

where *V* is the membrane potential, *C*_*m*_ the membrane capacitance and *I*_*ion*_ the sum of all the membrane currents. Details on each current are in the following subsections.

Properties of ion currents based on our recordings or derived from literature data on hESC-CMs were integrated into the model to reproduce Early and Late hESC-CM APs. Where data from hESC-CMs were not available, observations in ESC-derived or embryonic CMs from different species were considered. Although ionic channels undergo complex regulation at a transcript level, the I/V relationship of most currents does not change among different developmental stages [18-22]. Hence, we assumed that developmental changes in each current, I_xx_, are determined mainly by its quantitative change, which can be represented by setting a variable fraction (ratio, RaI_xx_) of the current maximal conductance in the adult model. Table 1 summarizes the maximal conductance values for the main currents in the model.

**Table 1:** Main developmental changes of ionic currents

	**Parameter (units)**	**Early**	**Late**	**Adult **[16]	**Species [Ref.]**
**I**_**to**_	G_to_ (S/F) max conductance	19.2929	48.702	294	human, EXP, †; human, MOD, [16]
**I**_**Kr**_	G_Kr_ (S/F) max conductance	288.0	134.4	96	rat, guinea pig, EXP, [24] ; human, MOD, [16]
**I**_f_^*2;^	G_f_ (S/F) max conductance	49	20.913	-	human, EXP, [6]
**I**_K1_^*2;^	G_K1_ (S/F) max conductance	240.523	1154.508	5405	human, EXP, [6] ; human, MOD, [16]
**I**_CaL_^*2;^	G_CaL_ (dm^3^/(F⋅s)) permeability	0.0438	0.0739	0.175	mouse, EXP, [24] ; human, EXP. [6] human, MOD, [16]
**I**_**CaT**_	G_CaT_ (S/F) max conductance	45.8	9.16	-	human, EXP, † ; rabbit, MOD, [27]
**I**_NaCa_^*2;^	I_maxNaCa_ (A/F) max current	17500	18240	1000	human, EXP, † ; human, MOD, [16]
**I**_**Na**_	G_Na_ (S/F) max conductance	563.844	14838	14838	mouse, EXP, [24] ; human, MOD, [16]
**I**_**Ks**_	G_Ks_ (S/F) max conductance	15.7	15.7	157	human, EXP, [32] ; human, MOD, [16]
**I**_**NaK**_	I_maxNaK_ (A/F) max current	0.9534	1.1305	1.362	mouse, EXP, [34] ; human, MOD, [16]
**I**_**pCa**_	G_pCa_ (S/F) max conductance	0.825, §	0.825, §	25	human, MOD, [16]
**I**_**up**_	I_maxUp_ (mM/s) max flux	0.0565	0.1403	0.425	mouse, EXP, [24] ; human, MOD, [16]
**I**_**rel**_	I_maxRel_ (mM/s) max flux	0.274	9.88	24.7	mouse, EXP, [24] ; human, MOD, [16]
**I**_**leak**_	I_maxLeak_ (1/s) max rate	0.0004	0.024	0.08	mouse, EXP, [24] ; human, MOD, [16]
**I**_**bCa**_	G_bCa_ (S/F) max conductance	0.118, §	0.592, §	0.592	human, MOD, [16]

Maximum conductances, currents and fluxes for Early and Late human embryonic stem cell-derived and adult ventricular cardiomyocyte models. The species considered for the Early and Late date are reported. Adult data refers to the TenTusscher model [16]. † new measurements and data; § values chosen to reproduce at best the AP shape; ^*2;^ not only the maximal conductance/current was changed but also the current formulation (see Additional file 1). EXP: experimental data; MOD: used for modelling.

#### Sodium current (I_Na_)

**Figure 1:**
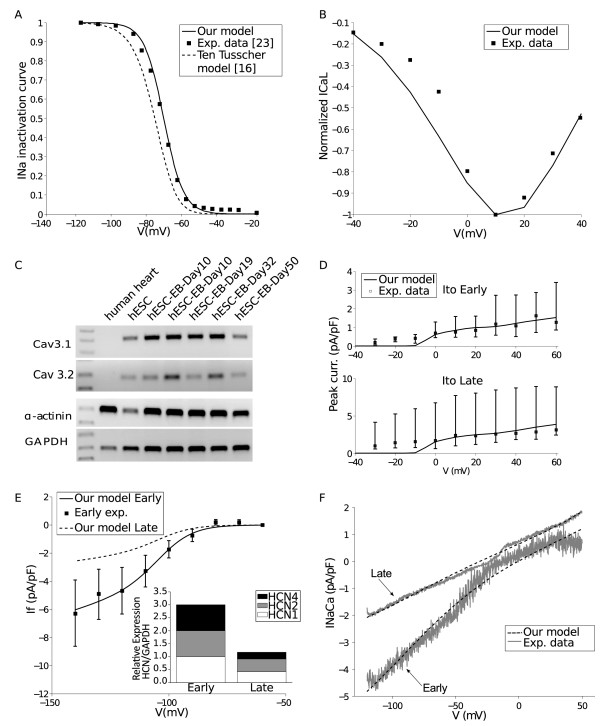
**Currents: experimental data and model fitting.** (**A**) I_Na_ inactivation for Early human embryonic stem cell-derived cardiomyocytes (hESC-CMs). (**B**) I_CaL_ normalized current-voltage (I/V) curve (Late stage). (**C**) Cav3.1, Cav3.2 and GADPH expression (I_CaT_) for H1-hESC-CMs. (**D**) I_to_ I/V curves. (**E**) I_f_ I/V curves and HCN quantitative expression (*inset*). (**F**) I_NaCa_ elicited by a voltage ramp protocol.

Our I_Na_ formulation slightly changes the original adult model (Additional file 1: Eqs. S3, S6 and S8, in Additional file 1). The steady-state inactivation was changed according to Satin et al. data on inactivation dynamics of H9.2-hESC-CMs at the Early stage of differentiation (Figure 1A) [23].

As also reported in [24] for rodent ESC-CMs we considered a small expression of I_Na_ at the Early stage and full expression at the Late one; the expression at the Early stage was further reduced with respect to [24] (from 0.08 to 0.038) in order to fit properly our experimental *maximal upstroke velocity* (Vmax) and the *action potential duration* (APD).

(2) $$RaI_{\mathrm{Na}}=0.038\;\left(\text{Early}\right);RaI_{\mathrm{Na}}=1\;\left(\text{Late}\right)$$

#### L-type calcium current (I_CaL_)

Due to lack of specific data on I_CaL_ at the Early stage, we tuned the permeability, the Ca^2+^ dependent inactivation gate, *f*_*Ca*_ (Additional file 1: Eqs. S25-S31), the time constant of the voltage dependent inactivation gate, *τ*_*f*_ (Additional file 1: Eq. S33), and the steady-state activation gate, *d*_*∞*_ (Additional file 1: Eq. S19) to reproduce the experimental features of AP, in particular APD.

We then slightly modified *d*_*∞*_ in order to fit our experimental recordings of I_CaL_ at Late developmental stage (Figure 1B, Late stage, n = 1), whereas inactivation parameters became equal to those of adult CMs in the Late stage formulation. We maintained the ratio between Late and Early conductances proposed in [24] based on data in mice and guinea pigs:

(3) $$RaI_{\mathrm{CaL}}=0.25\;\left(\text{Early}\right);RaI_{\mathrm{CaL}}=0.422\left(\text{Late}\right)$$

#### T-type calcium current (I_CaT_)

At variance with the adult model, we included I_CaT_, on the basis of different experimental evidence. First, I_CaT_ was reported to be highly expressed during fetal heart development and gradually decline after birth, becoming restricted to the conduction and pacemaker cells [25]. Secondly, I_CaT_ is functionally expressed in mouse ESC and is downregulated during cardiac differentiation: I_CaT_ channel subunits Cav3.1 and Cav3.2 expression decreased to approximately 46% and 24%, respectively, at 23.5 days of differentiation with respect to 9.5 days [26]. Finally, our qualitative RT-PCR measurements in H1-hESC-CMs show a clear expression of Cav3.1 and Cav3.2 at both stages (Figure 1C). We used the I_CaT_ formulation proposed in [27] in their sinoatrial node cell model, with progressively decreasing scaling factors for the maximal conductance:

(4) $$RaI_{\mathrm{CaT}}=0.25\;\left(\text{Early}\right);RaI_{\mathrm{CaT}}=0.05\left(\text{Late}\right)$$

#### Transient outward (I_to_), rapid and slow delayed rectifier (I_Kr_, I_Ks_) K^*+*^ currents

I_to_ properties were based on original data obtained in H1-hESC-CMs. Figure 1D shows the I/V relationship for peak K^+^ currents evoked by depolarizing steps in Early and Late CMs (median data and interquartile, n = 10 Early stage and n = 9 Late stage). A 10 mV positive shift was applied to experimental data to account for the use of Cd^2+^ to block I_CaL_, as done by [28]. According to our previous observations [6], I_to_ activation properties are similar to those described in native cardiac cells. Maximum conductances and steady-state activation were calculated by fitting experimental data (Figure 1D and Additional file 1: Eq. S43):

(5) $$RaI_{\mathrm{to}}=0.065622\;\left(\text{Early}\right);RaI_{\mathrm{to}}=0.165653\left(\text{Late}\right)$$

In accordance with various in vivo and in vitro experimental data in fetal guinea pigs [20] rats [29,30], and mice [31], I_Kr_ maximal conductance was greater than in the adult CMs and it decreased during maturation, we chose the specific expression ratios in order to mimick *maximum diastolic potential* (MDP) and the APD at the different developmental stages:

(6) $$RaI_{\mathrm{Kr}}=3\;\left(\text{Early}\right);RaI_{\mathrm{Kr}}=1.4\left(\text{Late}\right)$$

I_Ks_ conductance in the Early developmental stage was set in order to achieve a good fitting of the I/V curve (Additional file 1: Figure S1) obtained by [32] on Early hESC-CMs. On the basis of data on embryonic murine heart [19,24], where Early and Late developmental stages seem to share the same I_Ks_ conductance, a single value of RaI_Ks_ was used:

(7) $$RaI_{\mathrm{Ks}}=0.1\left(\text{Early and Late}\right)\text{;}$$

#### Inward rectifier K^*+*^ current (I_K1_)

In hESC-CMs I_K1_ is very small but not absent at the Early stage, then it increases during the development, as also reported for rat and guinea pig [24]. Conductances were identified according to our previous data [6], showing a ratio between Late and Early current density at −90 mV of 5.42. In order to reduce the MDP we set in our model a ratio of 4.8, within the variability of our data and introduced a shift of the voltage dependence for the inward rectification factor, x_k1∞_ (Additional file 1: Eqs. S67-S69), without modifying the current reversal potential.

(8) $$RaI_{K1}=0.0445\;\left(\text{Early}\right);RaI_{K1}=0.2136\left(\text{Late}\right)$$

#### Funny current (I_f_)

A key step of the formulation of a specific hESC-CM model consisted in integrating the hyperpolarization-activated cyclic nucleotide-gated or funny current, that is reported to be one of the main contributor for the spontaneous beating of pacemaker cells [33] and hESC-CMs [6]. I_f_ formulation (Additional file 1: Eqs. S71-S73) and conductance were obtained by fitting recordings performed on Early H1-hESC-CMs (Figure 1E, Early stage, n = 4).

For the Late stage, in accordance to our previous data [6], we assumed that current density decreased over maturation to an extent equal to the drop of cumulative HCN transcript expression (Figure 1E, inner panel): the estimated ratio between Early and Late stage was 2.34.

(9) $$RaI_{f}=0.5389\left(\text{Early}\right);RaI_{f}=0.23\;\left(\text{Late}\right)$$

#### Sodium-potassium pump (I_NaK_) and sodium-calcium exchanger (I_NaCa_)

Since data on hESC-CM I_NaK_ were not available, maximal current density was set taking into account its influence on the *diastolic depolarization rate* (DDR) and *frequency of spontaneous beating* (F) and reflecting the maturation related growth of INaK expression according to experiments by [34] on mouse ESC-CMs:

(10) $$RaI_{\mathrm{NaK}}=0.7\;\left(\text{Early}\right);RaI_{\mathrm{NaK}}=0.83\;\left(\text{Late}\right)$$

As far as I_NaCa_ is concerned, we used original experimental data from H1-hESC-CMs. Voltage ramp (from −120 to ^+^50 mV) protocols elicited an almost linear I_NaCa_ I/V relationship (Figure 1F, n = 6) that showed an inward and outward mode at both developmental stages. Fitting of experimental data led to modify the original maximal current density and the extra factor *α* in the I_NaCa_ expression [16]:

(11) $$\begin{array}{c} RaI_{\mathrm{NaCa}}=17.50\;\left(\text{Early}\right);RaI_{\mathrm{NaCa}}=18.24\;\left(\text{Late}\right)\\ a=0.8\;\left(\text{Early}\right),a=0.38\;\left(\text{Late}\right) \end{array}$$

#### Sarcoplasmic reticulum (SR) currents

The maximal values for the uptake (I_up_), release (I_rel_) and leakage (I_leak_) currents were tuned to simulate the ryanodine induced reduction of Ca^2+^ transient amplitude reported in [11] at the Early and in [12] at the Late stage. Increases in maximal current densities at the Late stage were based on the rodent ESC-CM model [24].

(12) $$\begin{array}{c} RaI_{\mathrm{Up}}=0.133\;\left(\text{Early}\right);RaI_{\mathrm{Up}}=0.33\;\left(\text{Late}\right)\\ RaI_{\mathrm{Rel}}=0.0111\;\left(\text{Early}\right);RaI_{\mathrm{Rel}}=0.4\;\left(\text{Late}\right)\\ RaI_{\mathrm{Leak}}=0.005556\;\left(\text{Early}\right);RaI_{\mathrm{Leak}}=0.3\;\left(\text{Late}\right) \end{array}$$

#### Sarcolemmal calcium pump, I_pCa_, and background current, I_bCa_

Since data about these currents are not available, we chose the following values to reproduce at best the AP shape:

(13) $$\begin{array}{c} RaI_{\mathrm{Cap}}=0.033\;\left(\text{Early and Late}\right);\\ RaI_{\mathrm{bCa}}=0.2\;\left(\text{Early}\right);RaI_{\mathrm{bCa}}=1\;\left(\text{Late}\right)\text{;} \end{array}$$

#### Cell capacitance and dimensions

Median values of measured cell membrane capacitance were used to set cell dimensions (see Additional file 1).

### Sensitivity analysis

A sensitivity analysis was performed according to the procedure reported in [35,36], opportunely adapted to our model. The main differences with respect to [35] are: (i) our hESC-CM model is not stimulated by an external source and (ii) we concentrate our analysis on the impact of the ratios RaI_xx_ on the AP shape. One ratio was varied at time by −20%, -10%, +10% and +20% respectively.

Considering the following ratios (parametrs) "p"

(14) $$\text{p}=\{{\text{RaI}}_{\text{Na}},{\text{RaI}}_{\text{CaL}},{\text{RaI}}_{\text{f}},{\text{RaI}}_{\text{to}},{\text{RaI}}_{\text{K}1},{\text{RaI}}_{\text{Kr}},{\text{RaI}}_{\text{Ks}},{\text{RaI}}_{\text{NaK}},{\text{RaI}}_{\text{NaCa}}\}$$

and the AP features (characteristics) "c"

(15) $$\text{c}=\left\{\text{MDP},\text{Vmax},\text{APD}30,\text{APD}50,\text{APD}70,\text{APD}90,\text{DDR},\text{F}\right\}\text{,}$$

computed after 300 seconds of simulation (assuming the steady state condition) the indexes percentage of change (D_c,p,a_), sensitivities (S_c,p,+20%_ and S_c,p,-20%_) and relative sensitivities (r_c,p,+20%_ and r_c,p,-20%_) were calculated as follows:

(16) $$D_{c,p,a}=\frac{\left(C_{p,a}+C_{\mathrm{control}}\right)}{C_{\mathrm{control}}}\cdot 100$$

(17) $$S_{c,p,+20\%}=\frac{D_{c,p,+20\%}}{0.2},S_{c,p,-20\%}=-\frac{D_{c,p,-20\%}}{0.2}$$

(18) $$r_{c,p,+20\%}=\frac{S_{c,p,+20\%}}{{\left|S_{c,p,+20\%}\right|}_{\max,c}},r_{c,p,-20\%}=\frac{S_{c,p,-20\%}}{{\left|S_{c,p,-20\%}\right|}_{\max,c}}$$

Splitting the original S_c,p_ and r_c,p_[35] was necessary since several tests resulted in no spontaneous APs, thus making impossible calculating the AP features and all their D_c,p,a_. However, for each ratio at least one D_c,p,a_ (D_c,p,+20% or_ D_c,p,-20%_) was available thus allowing to get the asymmetrical sensitivities.

### Interaction with in silico fibroblasts

In order to preserve intracellular milieu and cell-to-cell communication, AP recordings were not performed on single cells but on EBs, aggregates containing different cell phenotypes among which hESC-CMs and fibroblasts. To test the interaction between these kinds of cells and assess the effect on AP simulation, an additional mammalian fibroblast model, resistively coupled to the hESC-CM, was developed according to [37-39]. To this aim, the hESC-CM membrane potential equation was modified as follows:

(19) $$\frac{dV}{dt}=-{\frac{I_{\mathrm{ion}}+N_{f}\cdot I_{\mathrm{gap}}}{C_{m}}}_{\text{,}}$$

(20) $$I_{\mathrm{gap}}=G_{\mathrm{gap}}{\left(V-V_{\mathrm{fibro}}\right)}_{\text{,}}$$

where V_fibro_ is the fibroblast potential, G_gap_ is the conductance of the hESC-CM-fibroblast coupling (*G*_*gap*_*=* 1 *nS*) and N_f_ is the number of coupled fibroblasts. Details on the fibroblast model are reported in the Additional file 1 (Additional file 1: Eqs. S92-S94).

### Drug simulations

To reproduce the inhibition of SR Ca^2+^ release and the consequent Ca^2+^ transient reduction in ryanodine experiments the simulation was performed zeroing I_up_ and I_rel_. To simulate E4031 (I_Kr_ blocker) and nickel (I_CaT_, I_Kr_ and I_NaCa_ blocker) effects, in steady state conditions, a step reduction of conductance for the targeted currents was implemented in the model. The amount of conductance reduction for each current, which is reported in the Results Section, was chosen within the range of blocking action of the drug in order to better reproduce the specific experimental result obtained on a single cluster of hESC-CMs.

### Numerical implementation

Differential equations were implemented in MATLAB (The MathWorks, Natick, MA) and solved using ode15s.

## Results

### hESC-CM model

**Figure 2:**
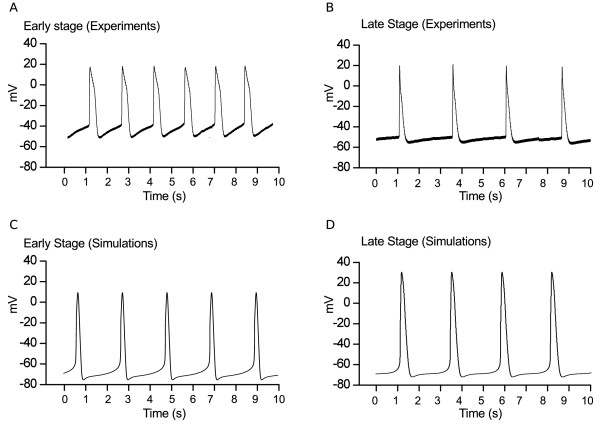
**Action Potential simulations.** Simulated action potentials for Early (**A**) and Late (**B**) hESC-CMs clusters.

Figure 2A shows simulated AP profiles for Early hESC-CMs obtained using our model: the modifications introduced with respect to the adult ventricular model were sufficient to elicit spontaneous beating. A comparison with experimental Early AP profiles obtained on intact EBs by multicellar recordings is provided in Table 2: a global comparison was done by calculating typical morphological parameters (AP features) for both experimental and simulated APs. This analysis demonstrated that our hESC-CM model was able to reproduce most of the experimental AP features, including *AP duration* (APD) at 30 (APD_30_), 50 (APD_50_), 70 (APD_70_) and 90% (APD_90_) of repolarization, Vmax, F and the DDR. Simulated and experimental data differed for the MDP, which is more negative in the simulation.

**Table 2:** Action potential features of experimental and simulated action potentials

	**Early**		**Late**		**Delta %**	
	**Exp**	**Sim**	**Exp**	**Sim**	**Exp**	**Sim**
APD _30_ (ms)	117 (107÷132)	121	163 (84÷256)	165	+ 40	+ 36
APD _50_ (ms)	169 (149÷184)	163	258 (148÷374)	224	+ 53	+ 37
APD _70_ (ms)	205 (181÷219)	191	325 (179÷465)	283	+ 59	+ 48
APD _90_ (ms)	229 (216÷241)	225	419 (223÷506)	350	+ 83	+ 56
Vmax (mV/s)	4610 (3308÷5612)	4123	5566 (5198÷7047)	5620	+ 42	+ 36
MDP (mV)	-55 (-62÷-37)	-76	-51 (-62÷-42)	-73	-7	- 4
DDR (mV/s)	14.4 (11.1÷28.4)	9.7	9.9 (8.0÷11.1)	7.1	- 31	- 27
F (bpm)	27 (23÷53)	29	22 (20÷29)	24	- 17	- 17

Delta %, Late VS Early; APD, action potential duration; V_max_ maximum upstroke velocity; MDP maximum diastolic potential; DDR diastolic depolarization rate; F frequency. Data reported as median and interquartile. All the experimental action potential features were measured on hESC-CM AP at the Early and Late stage [50].

### Transition from Early to Late stage of development: effects of maturation

The simulated AP profile at the Late stage and the comparison with experimental APs obtained on intact EBs by multicellar recordings are reported in Figure 2B and Table 2, respectively. These results show that the changes introduced in the model parameters between the Early and Late stage allow to reproduce the documented [6] maturation effects on AP shape. In particular, during the transition from the Early to the Late stage, APD and Vmax increase while spontaneous rate and slope of diastolic depolarization decrease.

### Spontaneous firing and action potential shape dependence on current conductances

The sensitivity analysis performed on the Early and Late models was aimed to assess how variations in the maximum conductances of the most important membrane currents affect (i) the phenomenon of spontaneous beating and (ii) the AP shape.

The spontaneous firing activity was not triggered at the Early stage in 2 tests only: RaI_CaL_ -20% and RaI_NaCa_ +20%. At the Late stage the spontaneous activity showed to be sensitive to more currents: the firing activity was triggered but stop after few dozens of seconds of simulations for RaI_Na_ +20%, RaI_CaL_-20%, RaI_f_ -20%, RaI_K1_ +20%, RaI_Kr_ +20%, RaI_NaK_ +20% and RaI_NaCa_ +20%.

**Figure 3:**
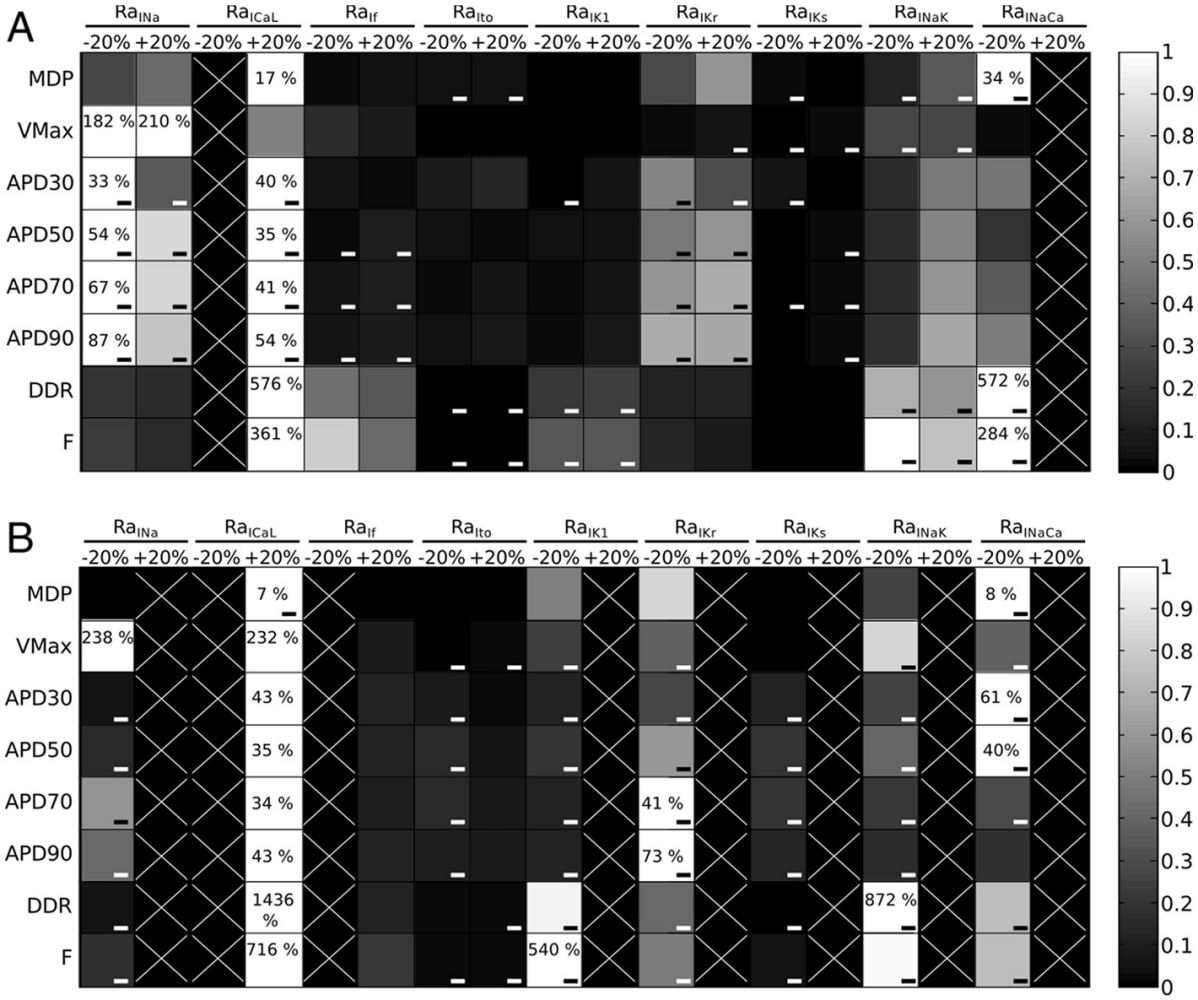
**Relative sensitivity maps for the Early (A) and Late (B) models.** AP features (rows) VS ratios RaI_xx_ (columns) used to rescale the maximum conductances/currents/fluxes were considered. For each RaI_xx_ the relative sensibilities at −20% and + 20% were taken into account. White color indicates maximum relative sensitivity of a particular AP feature among all ratios, whereas black indicates AP feature and ratio are independent. White X indicates the absence of spontaneous firing during a particular test. Percentages in each white box indicate the maximum absolute sensitivity of the AP feature correspondent to that row for all ratios. Negative sign indicates that AP feature and ratio vary inversely.

When the spontaneous beating allowed to reach a steady state condition, the AP features absolute/relative sensitivities were computed (Figure 3) as reported in the Methods section. At the Early stage MDP is affected by the inward I_CaL_, I_NaCa_ (inward during the late-repolarization) and the outward I_Kr_. The effect of I_f_ is extremely small, since at the MDP potential it is not activated yet, due to its high time constant. Also the I_K1_ effect is small since at this developmental stage the I_K1_ expression is low. The Late stage shows a more stable MDP (maximum variations: 7-8%), but the I_K1_ effect is stronger, as consequence of a maturation-related increment in I_K1_ expression. As expected, Vmax is mainly affected at both stages by the inward currents mainly acting during the upstroke: I_Na_ and I_CaL_, while the rate of spontaneous beating resulted to be more sensitive to I_CaL_, I_NaK_, I_NaCa_, I_f_ (Early stage)and I_K1_ (Late stage). I_CaL_, I_NaK_ and I_K1_ had the most strong effect on DDR and F at both stages; these AP features show also an important sensitivity to I_f_ increments at the Early stage. It is interesting to note that also I_NaCa_ reduction increase DDR and F at both stages but particularly at the Early one: a smaller I_NaCa_ (inward during late-repolarization) makes MDP more negative (−81 mV vs −76 mV, RaI_NaCa_-20% and control respectively) allowing a greater activation of I_f_ and thus increasing both DDR and F. Variations of the outward currents I_K1_ and I_NaK_ show the role of these currents in stabilizing the diastolic potential and, at the Late stage, increments block the spontaneous beating. APD is mainly affected by the inward currents I_CaL_ and I_Na_, by I_NaCa_ (outward during the upstroke), I_NaK_ (Late only) and the outward I_Kr_, especially during the late-repolarization. At the Early stage, the APD decreases after I_CaL_ increments: this counterintuitive result can be explained by using the model. In fact, it is due to a higher AP peak (20 mV vs 8.3 mV, RaI_CaL_ + 20% vs control): the higher reached membrane potential allows a stronger I_Kr_ activation thus reducing APD. At the Late stage this phenomenon is not present, since in the AP peak increment is smaller (34.2 mV vs 30.2 mV, RaI_CaL_ + 20% vs control), and I_Kr_ itself is smaller due to maturation. This different contribution of inward/outward currents to APD likely underlies the diverse APD rate-dependence (Additional file 1: Figure S2).

### Coupling with fibroblasts

**Figure 4:**
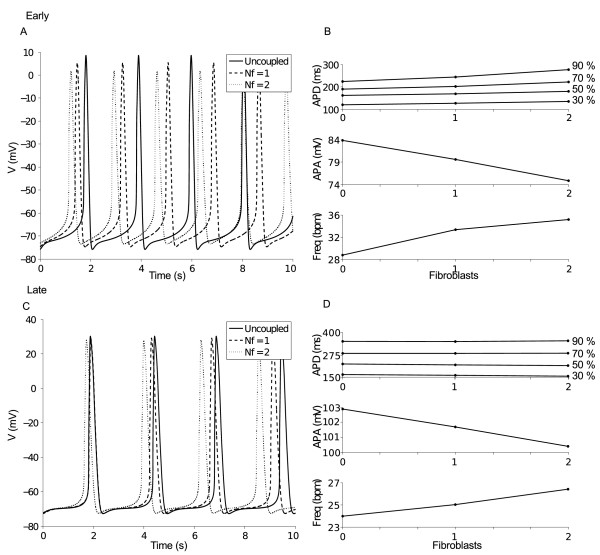
**Electrical coupling between human embryonic stem cell-derived cardiomyocyte and fibroblasts.** (**A**, **C**) Effects on action potentials (APs), Early and Late stage respectively, of a variable number (N_f_,) of fibroblasts. (**B**, **D**) Consequent changes of AP features, Early and Late stage respectively: both stages show an increment of the rate of spontaneous beating and an APA reduction. APD is slightly prolonged at the Early stage (especially APD70 and APD90) while at the Late stage the fibroblast effects are minor, showing only a slight reduction of APD30.

In order to partially overcome the discrepancy in MDP between the model and the experimental measurements, we introduced the contribution of fibroblasts, which are an essential component of EBs also for CM maturation. To assess the relevance of this issue in our model, we considered a simple system composed of a single hESC-CM coupled with 1 and 2 fibroblasts for each stage. Changes in basal AP due to fibroblast coupling are reported in Figure 4A and 4C, for the Early and Late phase, respectively, while a quantification of these changes is summarized in Figure 4B and 4D. Changes in most of the AP features as a function of fibroblast number were similar between the two stages. In particular, we observed an increment in DDR and rate while AP amplitude (APA) decreased. The effect on APD was different in the two stages. In the Early hESC-CM, lacking the plateau phase, the major effect is an inward current flowing from the coupled fibroblasts into the hESC-CM during the late repolarization phase, when hESC-CM membrane potential is negative to the fibroblast resting potential, promoting slowing of repolarization and APD increase. In the Late hESC-CM, showing significantly longer AP with respect to Early, the effect of an outward current, flowing towards coupled fibroblasts at depolarized potential and promoting hESC-CM repolarization and APD decrease, is also important. The overall result in Late hESC-CM is a decrease of APD_30_ and APD_50_ whereas APD_70_ and APD_90_ were almost not affected by coupling with fibroblasts. Importantly, as the number of coupled fibroblast increases from 0 to 2, a small rising of MDP occurred both at Early (−76 to −73 mV) and Late stage (−73 to -72 mV). This effect was accompanied by a reduction of Vmax at Early (4123 to 3443 mV/s) and Late stage (5620 to 2554 mV/s). At the same time, the membrane potential of coupled fibroblasts mimics the hESC-CM AP (data not shown), oscillating between −74 and 4 mV at the Early and between −72 and 26 mV at the Late stage.

### Intracellular calcium

To assess the relevance of RyR-mediated SR Ca^2+^ release in our model, we simulated cytoplasmic Ca^2+^ oscillations in control conditions and after blockade of Ca^2+^ release. In control conditions, the Early model showed intracellular Ca^2+^ diastolic and systolic concentrations of 0.026 μM and 0.141 μM, respectively, with an amplitude of the transient of 0.115 μM. The mean rate of decay was 0.123 μM/s while the maximum upstroke velocity (VmaxCa,upstroke) was 1 μM/s and the maximum decay velocity (VmaxCa,decay) was 0.52 μM/s. Control values for the Late model were 0.063 μM and 0.506 μM for diastolic and systolic concentrations, 0.443 μM and 0.340 μM/s for amplitude and mean rate of decay. VmaxCa,upstroke was 13 μM/s and VmaxCa,decay was 1.4 μM/s.

**Figure 5:**
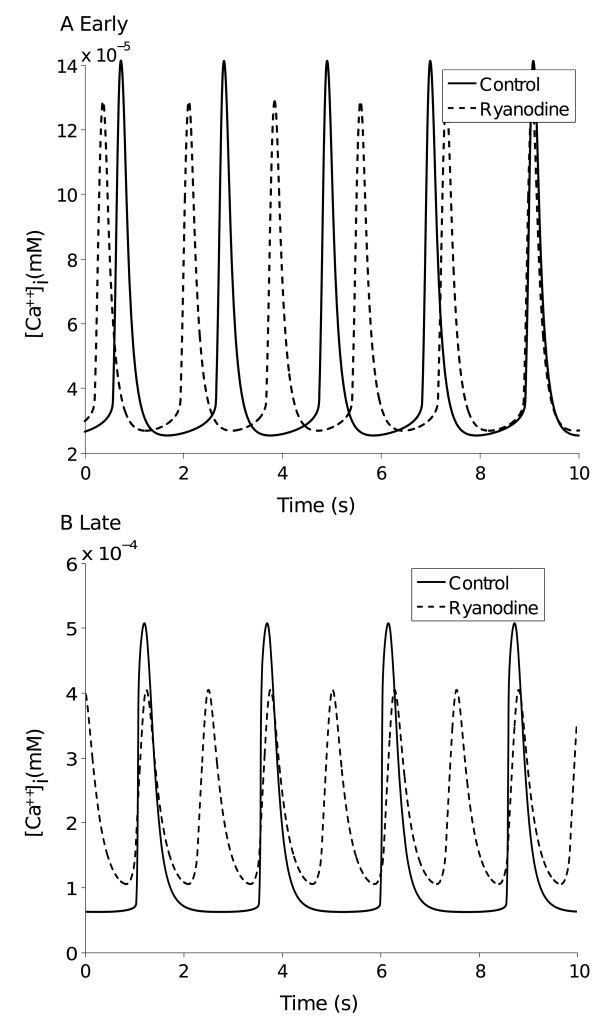
**Effects of Ryanodine.** Simulation of the reduction of calcium transient amplitude under the effect of Ryanodine at Early (**A**) and Late (**B**) stages.

The blockade of the SR channels and pumps reduced the Ca^2+^ oscillation amplitude by only 12% at the Early stage, whereas the RyR-induced reduction was larger, 33%, at the Late stage (Figure 5A and 5B). These results are similar to those reported experimentally in H1-CMs by [11] after 18-24 days and by [12] in cells assessed 30-40 days “post-beating”, likely corresponding to 50 days of total time of differentiation. A more detailed comparison was performed between the data reported in [11] on caffeine-sensitive H1-CMs and our Early stage, also considering the transient elicited in [11] in stimulation condition. In order to compare the experimental and simulated VmaxCa,upstroke and VmaxCa,decay in control conditions we normalized them using the amplitude of the transient, since experimental data were reported in terms of fluorescence while ours as concentration values. Estimating from [11] transient amplitude 0.16 F340/380 and VmaxCa,upstroke 1.5 F340/380/s we got a normalized VmaxCa,upstroke of 9.3 1/s. Normalizing our Early VmaxCa,upstroke, our model reproduced 8.7 1/s. Comparing in the same manner the VmaxCa,decay (estimated experimental VmaxCa,decay 0.3 F340/380/s) we got 1.9 1/s vs 4.5 1/s, respectively experimental [11] and simulated. The last comparison regards the reduced VmaxCa,upstroke value after RyR application: in [11] an experimental value of 70% was reported, while our model simulated 78%.

### Drug simulations

**Figure 6:**
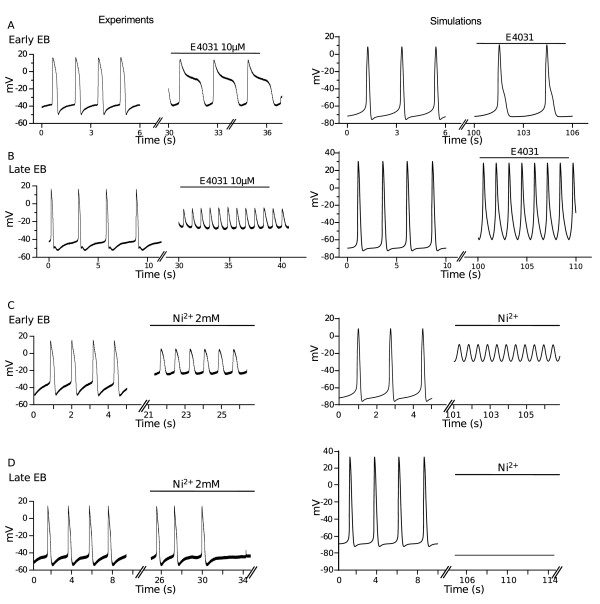
**Effects of current blocking with E4031 and Nickel.** Single experiments (*left*) and simulations (*right*) on the effect of 10 μM E4031 (I_Kr_ blocker) and of 2 mM Nickel (I_NaCa_, I_CaT_, I_Kr_ blocker) at Early (**A** and **C**) and Late (**B** and **D**) stages.

Finally, we performed a preliminar test of the qualitative reproduction of the main effects on AP of well-established channel blockers. E4031 is known to prolong cardiac AP through I_Kr_ selective blockade. We tested the effect of E4031 on a single spontaneously beating cluster of hESC-CMs at Early stage and observed a progressive decrease of the pacing frequency, a depolarization of MDP and an increase of APD involving all phases of repolarization (Figure 6A, *left*). These effects were fast on our experimental system and after 60 s from drug application the cluster showed a complete stop of its spontaneous electrical activity and beating [6]. To simulate this effect in our model, G_Kr_ was decreased by 50% of its Early value and this resulted in AP prolongation (Figure 6A *right*). In fact the AP lengthening measured at different values of repolarization were 12% (APD_30_), 18% (APD_50_), 47% (APD_70_) and 110% (APD_90_). In the simulation MDP was also depolarized by 4%, in accordance with a weaker contribution of repolarizing current, and frequency was reduced by 27%. In our model a decrease of I_Kr_ current larger than 50% produced a block of spontaneous beating, similarly to what observed experimentally [6].

In a single cluster of hESC-CMs at the Late stage, application of E4031 produced a fast depolarization of MDP and a remarkable increase in spontaneous rate with characteristic small amplitude oscillations of membrane potential (Figure 6B, *left*). At this stage, simulation was obtained by 60% reduction of G_Kr_ that resulted in AP modifications qualitatively similar, even if smaller, to those observed experimentally. In particular, rate was increased by 97%, APD_70_ by 19%, APD_90_ by 65%, MDP was depolarized by 17% (Figure 6B, *right*).

At millimolar concentration nickel is well known to largely block I_NaCa_ and I_CaT_. Furthermore, it has also been reported to block at a very high extent I_Kr_ in sinoatrial node cells [40]. All these currents are involved in different phases of spontaneous beating generation and AP. In the Early stage, application of nickel produced a marked MDP depolarization, a sustained APA reduction of and an increase of spontaneous rate, which lasted unaltered over time (Figure 6C, *left*). Conversely, at the Late stage, nickel first slowed down and then blocked completely electrical activity (Figure 6D, *left*). At both stages, simulations were performed reducing I_CaT_ and I_Kr_ by 90% and I_NaCa_ by 75% of their initial values. The effects on APA, MDP and beating rate detected at the Early stage were well reproduced (Figure 6C, *right*). Blockade of spontaneous activity observed at the Late stage was also clearly mimicked in our model (Figure 6D, *right*).

## Discussion

The major aim of this work was to develop a mathematical model able to reproduce the basal electrophysiological properties of hESC-CMs and the modifications induced by in vitro maturation. This approach, has been applied for the first time to hESC-CMs and the model represents the first computational tool for studying the fundamental physiology of hESC-CMs, in particular those derived from the H1 cell line. Moreover, introducing the contribution of fibroblasts we approached the simulation of the properties of beating intact EBs, which represent the elementary functional unit able to promote electrophysiological maturation of hESC-CMs and therefore suitable for screening ion channel blockers and assessing the cardiac safety of drugs.

We reached the goal of reproducing the spontaneous AP profile of hESC-CMs, strongly focusing on the modifications occurring during the transition of intact EBs from the Early to the Late developmental stage. Specifically, we reproduced key modifications documented experimentally on intact EBs by multicellular recordings, including the decrease of spontaneous AP frequency in association with a reduced DDR, meaning that the automaticity was slowed down during simulated maturation, similarly to experimental observations. The maturation-related increase of APD was also satisfactorily mimicked by our model, thus reflecting the modifications identified for many ionic currents occurring during in vitro maturation. Of note, we chose this specific electrophysiological approach on intact EBs due to several advantages, including the preservation of tissue architecture that allows the detection of single cell transmembrane voltage resulting also from the contribution of neighbouring cells. The latter, beside non myocytic cells, may be represented by CMs with similar or different phenotypes, with a relative composition of different phenotypes depending mainly from the developmental stage under evaluation. In fact Early hESC-CMs have a rather homogeneous phenotype (mostly sinus nodal like) that progresses into different phenotypes in the Late phase (atrial, ventricular and sinus nodal). Therefore, our experimental and simulation data readily reflect the degree of phenotype dishomogeneity in most of action potential parameters, resulting from the contribution of individual CMs at different stages (throughout the developmental process) and the fate (ventricular, atrial or nodal). To further confirm this statement, the median measurements of action potential duration at different percentage of repolarization have a lower interquartile range in the Early phase compared to the Late (see Table 2).

Based on the integration into our model of original and literature data on different ionic currents, some considerations can be drawn on their role in the hESC-CM development.

I_to_ is known to play a key role during the early repolarization phase leading to the notched shape and plateau phase present in human adult ventricular and atrial cells [41]. In our experimental conditions we found that I_to_ density increased from the Early to the Late phase. This result is in line with our previous data demonstrating a functional and molecular up-regulation of I_to_ during hESC-CMs maturation. It further confirms that this channel is a fundamental marker of functional CM maturation among mammalians [6]. These modifications were favourably integrated in our model, thus demonstrating their positive contribution to its final formulation. However, our measurements of I_to_ showed substantial current density also for negative potentials. For this reason I_to_ half-maximal activation was shifted from 20 to −5 mV (Figure 1D) whereas the steepness of the model curve was kept slightly greater than the experimental one, as already done and discussed in the adult model [16].

A different set of original data integrated in our model is related to I_f_. It is an inward current involved in the generation of spontaneous electrical activity, identified and characterized in our previous study on H1-hESC-CMs [6]. In the present study we further characterized this current identifying its functional relevance over an extended period of time. We calculated maximal conductance for the Early stage and extrapolated its value at the Late one on the basis of a cumulative reduction of HCN total transcript. Although functional properties of currents do not uniquely depend on the amount of channel transcript/s, we hypothesised that I_f_ density is likely to decrease during hESC maturation on the basis of different experimental evidences. First, the rate of spontaneous beating and the slope of the diastolic depolarization decrease with maturation (Table 1); secondly, I_f_ downregulation is a well established marker of functional maturation of native CMs [42,43]. These observations led us to integrate in our model a I_f_ contribution declining over maturation time. As observed for I_to_, this implementation had a positive effect on the model mimicking properties. As shown in Figure 3, modulation of I_f_ mainly affects in the model the rate of spontaneous beating and the DDR at both stages but especially at the Early one. The Late stage represented a scenario characterized by a smaller I_f_ and which evolves towards cells with no automaticity, so the 20% reduction of RaI_f_ led to no spontaneous activity.

Experimental evidence documents the occurrence of developmental changes of I_NaCa_ in different animal models, with maximal current density lowering throughout maturation [24]. In humans, I_NaCa_ expression peaks at mid-gestation, overcoming that of the adult heart [44]. In hESC-CMs, [11] reported that I_NaCa_ expression in the Early phase is higher with respect to that found in human fetal and adult ventricular CMs. This evidence is in line with our experimental results (see Figure 1F), showing larger current density both in Ca^2+^ outward and inward modes, at the Early stage with respect to the Late one. By contrast, a recent study [45] performed on a similar model led to opposite results, *i.e.* maximal current occurring in the Late stage. The explanation for such a difference is not obvious; diverse developmental window and/or cell phenotypes (ventricular and atrial vs ventricular) may account for these discrepancies. Model-based analysis showed that at both stages I_NaCa_ (Figure 3) modulation affects basically all the AP features (tests with RaI_NaCa_ -20%) and the occurrence of spontaneous beating (tests with RaI_NaCa_ +20%).

Other relevant maturation-related current modifications introduced in our model are consistent with previous observations in rodent ESC-CMs. In particular I_Kr_ decrease and I_Na_ increase confirmed to be important to achieve AP changes such as AP prolongation and increase in Vmax observed with hESC-CM development. As expected I_Na_ had the strongest influence on Vmax while I_Kr_ had a considerable effect on the APD, especially in the latest phases of the repolarization (see Figure 3).

The sensitivity analysis summarized in Figure 3 helps in assessing the maturation-related effects on the spontaneous contractile activity of hESC-CMs, in particular showing that at the Late stage it is more sensitive to current variations that at the Early stage. At the Early stage the reduction of the depolarizing I_CaL_ (necessary for the upstroke) and the increment of I_NaCa_ (inward current during the late-repolarization) caused the spontaneous beating stop. In addition to the reduced I_CaL_ and increased I_NaCa_ tests, the Late stage showed no spontaneous beating also for increments of the main repolarizing currents I_Kr_, I_K1_, I_Ks_ and I_NaK_, thus supporting the hypothesis of a weakening of the spontaneous depolarization during maturation. Moreover, also the effect of the 20% I_f_ reduction at the Late stage is indicative of this mechanism since I_f_ was already reduced during the transition from the Early to the Late stage.

We also tested coupling of fibroblasts with the hESC-CM model. Our hypothesis was that such approach could improve the quality of simulated APs that are generated by a complex system comprising CMs embedded in a cluster of different cells, such as fibroblasts. Indeed, one specific AP feature predicted by our model (MDP) differed substantially with respect to the experimental data. At both developmental stages, effects of coupling were small and consisted of MDP depolarization and increase of DDR and frequency. These effects can be explained by observing that during diastole fibroblast membrane potential is less negative with respect to that of CMs, causing a small depolarizing current flowing into CMs. Although limited, these modifications collectively move our simulation towards the experimental measurements, therefore improving the mimicking potential of our model. On this basis, it would be interesting to explore the possibility to enlarge the cell network by including different cell phenotypes (e.g. endothelial cells) possibly present in vivo. Globally, our results are in line with similar studies [37], where coupling of the TenTusscher model to fibroblasts led to a slightly depolarized resting potential, reduced APA and to the electrotonic modulation of the fibroblast potential by the coupled CM.

Recently, increasing attention has been focused on the development and maturation of the SR activity in hESC-CMs. Accumulated evidence points to a significant activity of Ca^2+^ release from SR [11,12], even if less organized and regulated than in mature CMs. In fact, while in the Early phase H1-hESC-CMs express SERCA2a, other proteins such as calsequestrin, triadin and junctin are almost absent [46]. Moreover, at 40-50 days of differentiation, T-tubules remain undetected on the sarcolemma, suggesting a topological environment different from mature, where Ca^2+^ influx through Ca^2+^ channels in the T-tubule is tightly associated with sarcoplasmic RyR channels [10]. Overexpression of calsequestrin in H1-hESC-CMs failed to induce the growth of T-tubules even though SR load and Ca^2+^ transient amplitude became more similar to those of mature CMs [47]. Simulations of Ca^2+^ transients using our model led to results fully consistent with the experimental data reported above. In fact, upon blockade of Ca^2+^ release from SR we found a decreased Ca^2+^ transient amplitude by 12% in the Early, and by 33% in the Late phase, similarly to the results reported experimentally [11,12]. Overall, these results indicate that in the Early stage Ca^2+^ cycling is mainly governed by sarcolemmal fluxes, while upon maturation Ca^2+^ release from SR increases its contribution moving toward a functional integration with sarcolemma to generate rhythmic activity. This evidence is in line with recent data obtained in late-stage mouse ESC [46] and further extends the predictive potential of our model to intracellular Ca^2+^ handling processes. Simulated block of Ca^2+^ release also increased the oscillation frequency (see Figure 5); we are not aware of experimental reports on this specific aspect in hESC-CMs which, on the other hand, seems in contrast with the effect of RyR knock out in ESC-CMs [48]. However, in the same conditions, a consistent increase in the frequency of AP-induced Ca^2+^ transients is apparent also in [12] (Figure 6), thus suggesting that further investigation supported also by model based analysis might provide mechanistic insights on this issue.

In our model, simulations of channel blockers were aimed to test, as a proof of concept, a qualitative reproduction of the main experimentally AP modifications observed in preliminar experiments. In the case of I_Kr_, a reduction in the maximal conductance simulated the application of E4031. This operation altered AP shapes quite similarly to what observed in the experiment, despite the fact that simulated AP prolongation was far less pronounced at the Early stage while at the Late stage MDP was more negative with respect to the experimental trace. Importantly, our model replicates the effect of E4031 on the frequency of spontaneous beating, which decreases in the Early stage and markedly increases in the Late one.

Our experiments using 2 mM nickel were simulated by a relevant blockade of I_NaCa_, I_CaT_ and I_Kr_ in the model, in accordance with the lack of selectivity of nickel at this concentration and the high levels of block reported for these currents. At the Early stage simulations and experimental recording similarly show a residual electrical activity slightly different in frequency and amplitude (see Figure 6C); nonetheless, both results suggest that residual currents are sufficient to drive a repetitive potential oscillation of small amplitude occurring at depolarized potentials. For the Late stage experimental traces and simulations show a complete block of spontaneous activity (see Figure 6D) occurring at negative potential values, opening the hypothesis that in this phase, where other depolarizing currents such as I_f_ are downregulated, I_NaCa_ plays a key role in sustaining the spontaneous activity. Our results are consistent with those of similar experiments [45] performed with 5 mM nickel, a concentration expected to exert a higher level of channel blockade. In fact, with this higher nickel concentration no Ca^2+^ transients due to spontaneous activity were recorded at any developmental stage.

### Limitations

The main limitations of our work are related to i) the shortage of data and ii) the variability of phenotypes. We built an AP model for human embryonic stem cells derived cardiomyocytes. However, many data to build the model are taken from published experimental work on different animal species. The crossbreeding process of data from human and animal CMs represented a compulsory choice for our model, since some current properties expressed in the adult ventricular cells were unknown or not sufficiently characterized in hESC-CMs. A more accurate numerical model of “human” embryonic stem cells derived cardiomyocytes would require further experimental investigations. This should be considered as a first step in the effort of mathematically describing the ESC-CM electrophysiology aimed at capturing the qualitative mechanisms.

The choice of a specific human adult ventricular AP model as the basis for the hESC-CM model could be perceived as a limitation of the present work. We chose as the “starting point” for the development of our model a modified version of the TenTusscher model [15], in which I_Kr_, I_Ks_ and I_CaL_ were changed with respect to their original formulation. From this starting point, in order to reproduce developmental changes in each current we modulated (through the RaI_xx_ ratios) the current maximal conductances. But at the same time the kinetic of several currents were changed based on data from ESC-CMs: I_Na_, I_CaL_, I_to_, I_K1_, I_NaCa_. Moreover, two relevant membrane currents that were not present in the adult ventricular model (I_CaT_ and I_f_) were incorporated in our model. All these modifications reduced the actual impact on the final results of the initial choice of the starting point. A sensitivity analysis with respect to the choice of the parent model was far beyond the scope of the present work. It is also worth noting that the modification of the Ten Tusscher model allowed the correct prediction of APD shortening at higher [Ca^2+^_o_[15]. All the more recent models (e.g. [49]) show unrealistic APD prolongation upon increase of extracellular calcium. This aspect is particularly relevant for our study, in which we had to reproduce intracellular AP recordings obtained with 2.7 mM Ca^2+^ in the external solution [50].

The difference in MDP between experiments and simulations is a limitation, possibly related to an incomplete description of the complex interconnection within the EBs. For example, in simulating AP recorded from EBs, only CM to fibroblast coupling has been considered. Other variables, such as CM to CM gap junctions likely contribute to the AP properties of EBs. However, such a term would dramatically complicate the system of differential equations and goes beyond the scope of the present work.

## Conclusions

In conclusion, this study provides the first modelling tool able to simulate the membrane AP, the associated intracellular Ca^2+^ handling properties and the modification occurring over the maturation process of hESC-CMs. The simulation of the transition from Early to Late developmental stage involved: increasing I_to_ density, declining I_f_ contribution, decreasing I_NaCa_ current density, I_Kr_ decrease and I_Na_ increase. Moreover, an increasing contribution to Ca^2+^ cycling of the Ca^2+^ release from SR was pointed out.

We expect to overcome inherent limitations present in the model by further experimental investigations exploring unknown properties of basic physiology of hESC-CMs, possibly including different stem cell lines. Also, the combined use of novel pharmacological/simulated challenges will be useful to implement and validate the predictive potential of the model. Finally, novel challenges come from studies (including drug testing) in CMs from induced pluripotent stem cells carrying genetic mutations [51,52]; the development of disease-specific cell lines for genetic cardiac disorders prompts toward the refinement of this mathematical modelling to address future directions in this field.

## Abbreviations

AP: Action potential; APA: Action potential amplitude; APD: Action potential duration; APD_XX_: Action potential duration at XX% of repolarization; C_m_: Membrance capacitance; CM: Cardiomyocyte; DDR: Diastolic depolarization rate; EB: Embryoid body; F: Frequency of spontaneous beating; G_xx_: I_xx_ current conductance; hESC: Human embryonic stem cell; hESC-CM: Human embryonic stem cell-derived cardiomyocyte; I_bCa_: Background Ca^2+^ current; I_CaL_: L-type Ca^2+^ current; I_CaT_: T-type Ca^2+^ current; I_f_: Hyperpolarization activated funny current; I_Kr_: Rapid delayed rectifying K^+^ current; I_Ks_: Slow delayed rectifying K^+^ current; I_K1_: Inward rectifying K^+^ current; I_leak_: Leakage Ca^2+^ current; I_maxXX_: I_xx_ maximal current; I_Na_: Na^+^ current; I_NaCa_: Na^+^/Ca^2+^ exchanger current; I_NaK_: Na^+^/K^+^ pump; I_pCa_: Sarcolemmal Ca^2+^ pump; I_rel_: Release Ca^2+^ current; I_to_: Transient outward K^+^ current; I_up_: Uptake Ca^2+^ current; MDP: Maximum diastolic potential; Ra_Ixx_: Variable fraction of the I_xx_ current maximal conductance in the adult model; SR: Sarcoplasmic reticulum; Vmax: Maximal upstroke velocity of the membrane potential; VmaxCa,upstroke: Maximal upstroke velocity of the Ca^2+^ transient; VmaxCa,decay: Maximal decay velocity of the Ca^2+^ transient.

## Competing interests

The authors declare that they have no competing interests.

## Authors’ contributions

MP: conception and design, data analysis and interpretation, manuscript writing, final approval of manuscript. LS: provision of study material, collection and assembly of data, data analysis and interpretation, manuscript writing, final approval of manuscript. MDL: provision of study material, collection and assembly of data, manuscript revision, final approval of manuscript. MJ: provision of study material, manuscript revision, final approval of manuscript. AM: provision of study material, manuscript revision, final approval of manuscript. EC: conception and design, collection and assembly of data, data analysis and interpretation, final approval of manuscript. SS: conception and design, data analysis and interpretation, manuscript writing, final approval of manuscript. All authors read and approved the final manuscript.

## Supplementary Material

Additional file 1 **Supplementary Methods: Mathematical Modelling of the Action Potential of Human Embryonic Stem Cell derived Cardiomyocytes. Additionalfigure S1**: I_Ks_ tail current. **Additional figure S2**: APD rate-dependence [53,54]. Click here for file
