# Supplementary material for: Mathematical modelling of the action potential of human embryonic stem cell derived cardiomyocytes
Source: Biomed Eng Online. 2012 Aug 28;11:61. doi: 10.1186/1475-925X-11-61 (PMC3477113; doi:10.1186/1475-925X-11-61)
Supplement: Additional file 1 — Supplementary Methods: Mathematical Modelling of the Action Potential of Human Embryonic Stem Cell derived Cardiomyocytes. Additionalfigure S1: IKs tail current. Additional figure S2: APD rate-dependence [53,54]. [file 1475-925X-11-61-S1.pdf]

Supplementary Methods: Mathematical Modelling of the Action Potential of Human Embryonic Stem Cell derived Cardiomyocytes

Michelangelo Paci, Laura Sartiani, Martina Del Lungo, Marisa Jaconi, Alessandro Mugelli, Elisabetta Cerbai, Stefano Severi

Human ESC culture and differentiation

The human embryonic stem cell (hESC) line H1 from WICELL RESEARCH INSTITUTE (Madison, WI) was cultivated following WICELL protocols. Briefly, undifferentiated hESCs were grown on irradiated mouse embryonic fibroblasts and passaged once a week using collagenase and mechanical dissociation. Propagation medium was composed of DMEM/F12 supplemented with 20% Serum Replacement, 1% penicillin-streptomycin, 1% non-essential amino-acids (NEAA), b-mercaptoethanol (SIGMA-ALDRICH CHEMIE, Buchs, Switzerland), L-Glutamine and 4 ng/ml human bFGF. Embryoid bodies (EBs) formation was obtained from hESC colonies incubated with collagenase and gently scraped in differentiation medium (KnockOut-DMEM supplemented with 20% Defined FBS (HYCLONE LABORATORIES South Logan, UT), 1% penicillin-streptomycin, 1% NEAA, L-Glutamine and b-mercapto-ethanol). EBs were cultured for 4 days in Costar ultra-low attachment 6-well plates (CORNING, Schiphol-Rijk, The Netherlands), with medium change every 2 days. EBs were then plated on gelatin-coated 6 cm-dishes and medium was changed every 2-3 days.

Cell isolation

Beating clumps were dissected from embryo bodies using a microscalpel and directly placed into the solution used to perform intracellular recordings. Alternatively, they were digested with 1 mg/ml collagenase B (ROCHE, Basel, Switzerland) in PBS supplemented with 30 mM calcium for 15-20 minute at $37°C$ with pipetting every 5-10 min. Dissociated cells were then plated on gelatin-coated dishes in differentiation medium and used for patch-clamp recordings.

Patch-clamp recordings

The experimental set-up for patch-clamp (whole-cell) recording and data acquisition was similar to that described previously [1]. Briefly, the patch-clamped cell was superfused by means of a temperature-controlled ($37°C$) micro-superfusor allowing rapid changes of the solution bathing the cell. Patch-clamp pipettes, prepared from glass capillary tubes (HARVARD APPARATUS, Edenbridge, UK) by means of a two-stage horizontal puller (SUTTER INSTITUTE, Sacramento, CA, model P-87), had a resistance of 2-3 MW when filled with the internal solution. Cell membrane capacitance (C_m) was measured by integrating the capacitance current recorded during a ±10 mV hyperpolarizing pulse from a holding potential of -70 mV, as previously reported [1]. Series resistance and membrane capacitance were compensated in order to minimize the capacitive transient. Transient outward potassium current (I_{to}) was evoked by steps from –40 to +70 mV (holding potential –70 mV), after a pre-step to –40 mV to inactivate sodium current (I_{Na}). I_{to} was measured as the difference between peak outward current at the beginning of the depolarizing step and the steady state current at the end of the step, and normalized with respect to C_m. The funny current (I_f) was evoked by hyperpolarizing steps from -50 to -130 mV (holding potential –40 mV). Steady-state values of current were calculated by fitting current traces to a mono-exponential function. I_f amplitudes were measured as the difference between the extrapolated value at the steady state and that at the beginning of the test pulse, and normalized with respect to C_m, as reported elsewhere [2]. Sodium-calcium exchanger (I_{NaCa}) current was elicited with an ascending voltage ramp (from -120 to

+70 mV, holding potential -40mV). Current was defined as the difference current in the absence and presence of $NiCl_2$ (2 mM) and amplitude was normalized to whole cell capacitance.

Intracellular recordings

The experimental set-up for intracellular recordings and data acquisition was similar to that described previously [2]. Briefly, spontaneously beating EBs were fixed on bottom of a perfusion chamber, thermostatically controlled at $33 - 35°C$ and superfused a constant-flow. Embryoid body electrical activity was recorded using a standard electrophysiological techniques, as previously reported [3]. Briefly, the recording electrode consisted of a short Ag/AgCl pin that was partly inserted into a floating glass microelectrode containing 3 M KCl and connected to the headstage of the amplifier. An Ag/AgCl pellet served as reference electrode in the perfusion chamber. The tip resistance of the microelectrode ranged between 30 and 40 MW. The recording microelectrode and the reference electrode were connected through a high input impedance amplifier (BIOMEDICA MANGONI, Pisa, Italy) interfaced with a computer. The microelectrode was slowly moved into the chamber under microscopic inspection with the use of a micromanipulator. The electrode potential was compensated to zero in the bathing solution. Spontaneous APs were digitized by an A/D converter and analyzed off-line with Iox software (EMKA TECHNOLOGY, Falls Church, VA).

Solutions

Normal Tyrode's solution (in mM): $NaCl$ 140; KCl 5.4; $CaCl_2$ 1.8; $MgCl_2$ 1.2; $D-glucose$ 10; $HEPES$ 5 (pH 7.35 with $NaOH$).
Modified Tyrode's solution for I_f current (in mM): $NaCl$ 140, KCl 25, $CaCl_2$ 1.5, $MgCl_2$ 1.2, $BaCl_2$ 2, $MnCl_2$ 2, $4 - aminopyridine$ 0.5, $glucose$ 10, $HEPES - NaOH$ 5 (pH 7.35); this solution allowed the reduction of interference from other currents, i.e. I_{CaL} , I_{CaT}, I_{K1} and I_{to}. Modified Tyrode's solution for I_{to} current: normal Tyrode's solution plus $CdCl_2$ 0.5 mM.
Pipette solutions for AP, I_{to}, I_f (in mM): $K - Aspartate$ 130; $Na_2 - ATP$ 5, $MgCl_2$ 2, $CaCl_2$ 5, $EGTA$ 11, $HEPES-KOH$ 10 (pH 7.2). Pipette solutions for I_{CaL} (in mM): $Mg-ATP$ 5, $EGTA$ 15, $TEA-Cl$ 20, $HEPES$ 10, $CsCl$ 125, (pH 7.20 with $CsOH$).
Solution for I_{NaCa} current (in mM): $NaCl$ 128; $CsCl$ 10; $CaCl_2$ 2; $MgCl_2$ 1; $Na-HEPES$ 10; $Glucose$ 10; $Lacidipine$ 10; $SITS$ 100 ; $Ouabaine$ 0.5. (PH 7.4 with $CsOH$); this solution allowed the reduction of interference from other currents, i.e. I_{CaL}, I_{Cl}, I_{NaK}.
Pipette solutions for I_{NaCa} (in mM): $CsCl$ 120; $CaCl_2$ 3; $MgCl_2$ 0.5; $HEPES$ 20; $Mg - ATP$ 5; $BAPTA(K^+)$ 4 5; (PH 7.25 with $CsOH$)
External solution for intracellular recordings (in mM): $NaCl$ 125, KCl 4, $NaHCO_3$ 25, NaH_2PO_4 0.5, $MgSO_4$ 1.2, $CaCl_2$ 2.7, $Glucose$ 1 (pH 7.2 when gassed with 5% CO_2/95%O_2.
Pipette solution for intracellular recordings (in mM): KCl 3

Formulation of the hESC-CM model

Model Parameters

RaI_{xx} represents a variable fraction (ratio), of the current maximal conductance in the adult model ([4], except where differently specified).

$$Na_o = 150.5\ (mM)$$

$$K_o = 4\ (mM)$$

$$Ca_o = 2.7\ (mM)$$

$$Na_i = 7\ (mM)$$

$$K_i = 140\ (mM)$$

$$Ca_i = 0.0002\ (mM)$$

$$Ca_{SR} = 0.2\ (mM)$$

$$g_{CaL} = 0.000175 \times Ra_{ICaL}\ (m^3/(F \times s))$$

$$Ra_{ICaL} = \begin{cases} 0.25 & , Early \\ 0.422 & , Late \end{cases}\ (dimensionless)$$

$$g_{bca} = 0.592\ \times Ra_{Iback} (S/F)$$

$$Ra_{Iback} = \begin{cases} 0.2 & , Early \\ 1 & , Late \end{cases}\ (dimensionless)$$

$$Buf_c = 0.25\ (mM)$$

$$Buf_{sr} = 10\ (mM)$$

$$K_{buf_c} = 0.001\ (mM)$$

$$K_{buf_{sr}} = 0.3\ (mM)$$

$$K_{up} = 0.00025\ (mM)$$

$$V_{leak} = 0.08\ \times Ra_{Ileak}\ (1/s)$$

$$Ra_{Ileak} = \begin{cases} 0.005556 & , Early \\ 0.3 & , Late \end{cases}\ (dimensionless)$$

$$Cm = 185\ \times Ra_{Cm}\, (pF) = \begin{cases} 41\ pF, Early\ (30\ cells) \\ 33\ pF,\ Late\ (26\ cells) \end{cases}$$

$$Ra_{Cm} = \begin{cases} 0.22162 & , Early \\ 0.17838 & , Late \end{cases}\ (dimensionless)$$

$$V_c = 0.016404 \times Ra_{Cm}\ (m^3)$$

$$V_{sr} = 0.001094\ \times Ra_{Cm}\ (m^3)$$

$$Vmax_{up} = 0.425 \times Ra_{Iup}\ (mM/s)$$

$$Ra_{Iup} = \begin{cases} 0.133 & , Early \\ 0.33 & , Late \end{cases}\ (dimensionless)$$

$$a_{rel} = 16.464\ (mM/s)$$

$$b_{rel} = 0.25\ (mM)$$

$$c_{rel} = 8.232\ (mM/s)$$

$$Ra_{Irel} = \begin{cases} 0.0111 & , Early \\ 0.4 & , Late \end{cases}\ (dimensionless)$$

$$\tau_g = 2\ (ms)$$

$$K_{pCa} = 0.0005\ (mM)$$

$$g_{pCa} = 0.825 \times Ra_{IpCa}(A/F)$$

$$Ra_{IpCa} = 1 \ (dimensionless)$$

$$g_{Na} = 14838 \times Ra_{INa}(S/F)$$

$$Ra_{INa} = \begin{cases} 0.038 & , Early \\ 1 & , Late \end{cases} \quad (dimensionless)$$

$$g_{K1} = 5405 \times Ra_{IK1} \ (S/F)$$

$$Ra_{IK1} = \begin{cases} 0.0445 & , Early \\ 0.2136 & , Late \end{cases} \quad (dimensionless)$$

$$F = 96485.3415 \ (C \times mM)$$

$$R = 8.314472 \ (J/(M \times K))$$

$$T = 310 \ (K)$$

$$g_{Kr} = 96 \times Ra_{IKr}(S/F)$$

$$Ra_{IKr} = \begin{cases} 3 & , Early \\ 1.4 & , Late \end{cases} \quad (dimensionless)$$

$$L_0 = 0.025 \ (dimensionless)$$

$$P_{kna} = 0.03 \ (dimensionless)$$

$$g_{Ks} = 157 \times Ra_{IKs} \ (S/F)$$

$$Ra_{IKs} = 0.1 \ (dimensionless)$$

$$K_{NaCa} = 1000 \times Ra_{INaCa}(A/F)$$

$$Ra_{INaCa} = \begin{cases} 17.50 & , Early \\ 18.24 & , Late \end{cases} \quad (dimensionless)$$

$$K_{sat} = 0.1 \ (dimensionless)$$

$$Km_{Ca} = 1.38 \ (mM)$$

$$Km_{\ ai} = 87.5 \ (mM)$$

$$\alpha = \begin{cases} 0.8 & , Early \\ 0.38 & , Late \end{cases} \quad (dimensionless)$$

$$\gamma = 0.35 \ (dimensionless)$$

$$K_{mNa} = 40 \ (mM)$$

$$K_{mk} = 1 \ (mM)$$

$$P_{NaK} = 1.362 \times Ra_{INaK}(A/F)$$

$$Ra_{INaK} = \begin{cases} 0.7 & , Early \\ 0.83 & , Late \end{cases} \quad (dimensionless)$$

$$g_{to} = 294 \times Ra_{Ito} \ (S/F)$$

$$Ra_{Ito} = \begin{cases} 0.065622 & , Early \\ 0.165653 & , Late \end{cases} \quad (dimensionless)$$

$$g_f = 90.926 \times Ra_{If} \ (S/F)$$

$$Ra_{If} = \begin{cases} 0.5389 & , Early \\ 0.23 & , Late \end{cases} \quad (dimensionless)$$

$$\tau_f = 1900 \ (ms)$$

Membrane Potential

$$\frac{\mathrm{d}V}{\mathrm{d}time} = -I_{ion} = -\left(I_{K1} + I_{to} + I_{Kr} + I_{Ks} + I_{CaL} + I_{NaK} + I_{Na} + I_{NaCa} + I_{b_{Ca}} + I_{p_{Ca}} + I_{CaT} + I_f\right) \quad \text{(S1)}$$

Na^+ current, I_{Na}

$$I_{Na} = g_{Na} \cdot m^3 \cdot h \cdot j \cdot (V - E_{Na}) \quad \text{(S2)}$$

I_{Na}, h gate

$$h_{inf} = \begin{cases} \frac{1}{\sqrt{\left(1+e^{\frac{V+73}{5.6}}\right)}} & ,Early \\ \frac{1}{\left(1+e^{\frac{V+71.55}{7.43}}\right)^2} & ,Late \end{cases} \quad \text{(S3)}$$

$$\alpha_h = \begin{cases} 0.057 \cdot e^{\frac{-(V+80)}{6.8}}, \text{ if } V < -40 \\ 0, \text{ otherwise} \end{cases} \quad \text{(S4)}$$

$$\beta_h = \begin{cases} 2.7 \cdot e^{0.079 \cdot V} + 3.1 \times 10^5 \cdot e^{0.3485 \cdot V}, \text{ if } V < -40 \\ \frac{0.77}{0.13 \cdot \left(1+e^{\frac{V+10.66}{-11.1}}\right)}, \text{ otherwise} \end{cases} \quad \text{(S5)}$$

$$\tau_h = \frac{2.8}{\alpha_h + \beta_h} \quad \text{(S6)}$$

$$\frac{\mathrm{d}h}{\mathrm{d}time} = \frac{h_{inf} - h}{\tau_h} \quad \text{(S7)}$$

I_{Na}, j gate

$$j_{inf} = \begin{cases} \frac{1}{\sqrt{\left(1+e^{\frac{V+73}{5.6}}\right)}} & ,Early \\ \frac{1}{\left(1+e^{\frac{V+71.55}{7.43}}\right)^2} & ,Late \end{cases} \quad \text{(S8)}$$

$$\alpha_j = \begin{cases} \frac{\left(-25428 \cdot e^{0.2444 \cdot V} - 6.948 \times 10^{-6} \cdot e^{-0.04391 \cdot V}\right) \cdot (V+37.78)}{\frac{1}{1+e^{0.311 \cdot (V+79.23)}}}, \text{ if } V < -40 \\ 0, \text{ otherwise} \end{cases} \quad \text{(S9)}$$

$$\beta_j = \begin{cases} \frac{0.02424 \cdot e^{-0.01052 \cdot V}}{1+e^{-0.1378 \cdot (V+40.14)}}, \text{ if } V < -40 \\ \frac{0.6 \cdot e^{0.057 \cdot V}}{1+e^{-0.1 \cdot (V+32)}}, \text{ otherwise} \end{cases} \quad \text{(S10)}$$

$$\tau_j = \frac{1}{\alpha_j + \beta_j} \quad \text{(S11)}$$

$$\frac{\mathrm{d}j}{\mathrm{d}time} = \frac{j_{inf} - j}{\tau_j} \quad \text{(S12)}$$

I_{Na}, m gate

$$m_{inf} = \frac{1}{\left(1+e^{\frac{-56.86-V}{9.03}}\right)^2} \quad \text{(S13)}$$

$$\alpha_m = \frac{1}{1+e^{\frac{-60-V}{5}}} \quad \text{(S14)}$$

$$\beta_m = \frac{0.1}{1+e^{\frac{V+35}{5}}} + \frac{0.1}{1+e^{\frac{V-50}{200}}} \quad \text{(S15)}$$

$$\tau_m = \alpha_m \cdot \beta_m \quad \text{(S16)}$$

$$\frac{\mathrm{d}m}{\mathrm{d}time} = \frac{m_{inf} - m}{\tau_m} \quad \text{(S17)}$$

L-type Ca^{2+} current, I_{CaL}

$$I_{CaL} = \frac{g_{CaL} \cdot d \cdot f \cdot fCa \cdot 4 \cdot V \cdot F^2}{R \cdot T} \cdot \frac{\left(Ca_i \cdot e^{\frac{2 \cdot V \cdot F}{R \cdot T}} - 0.341 \cdot Ca_o\right)}{e^{\frac{2 \cdot V \cdot F}{R \cdot T}} - 1} \tag{S18}$$

I_{CaL}, *d* gate

$$d_{inf} = \begin{cases} \frac{1}{1+e^{\frac{12.5-V}{12.5}}} & ,Early \\ \frac{1}{1+e^{\frac{16-V}{12.8}}} & ,Late \end{cases} \tag{S19}$$

$$\alpha_d = \frac{1.4}{1+e^{\frac{-35-V}{13}}} + 0.25 \tag{S20}$$

$$\beta_d = \frac{1.4}{1+e^{\frac{V+5}{5}}} \tag{S21}$$

$$\gamma_d = \frac{1}{1+e^{\frac{50-V}{20}}} \tag{S22}$$

$$\tau_d = \alpha_d \cdot \beta_d + \gamma_d \tag{S23}$$

$$\frac{\mathrm{d}d}{\mathrm{d}time} = \frac{d_{inf} - d}{\tau_d} \tag{S24}$$

I_{CaL}, f_{Ca} gate [5]

$$\alpha_{fCa} = \begin{cases} \frac{1}{1+\left(\frac{Ca_i+0.00011}{0.000325}\right)^8} & ,Early \\ \frac{1}{1+\left(\frac{Ca_i}{0.0006}\right)^8} & ,Late \end{cases} \tag{S25}$$

$$\beta_{fCa} = \begin{cases} \frac{0.1}{1+e^{\frac{Ca_i+0.00011-0.0005}{0.0001}}} & ,Early \\ \frac{0.1}{1+e^{\frac{Ca_i-0.0009}{0.0001}}} & ,Late \end{cases} \tag{S26}$$

$$_{fCa} = \begin{cases} \frac{0.2}{1+e^{\frac{Ca_i+0.00011-0.00075}{0.0008}}} & ,Early \\ \frac{0.3}{1+e^{\frac{Ca_i-0.00075}{0.0008}}} & ,Late \end{cases} \tag{S27}$$

$$fCa\ _{nf} = \begin{cases} \frac{\alpha_{fCa}+\beta_{fCa}+_{fCa}+0.23}{1.46} & ,Early \\ \frac{\alpha_{fCa}+\beta_{fCa}+_{fCa}}{1.3156} & ,Late \end{cases} \tag{S28}$$

$$\tau_{fCa} = 2 \tag{S29}$$

$$d_{fCa} = \frac{fCa_{inf} - fCa}{\tau_{fCa}} \tag{S30}$$

$$\frac{\mathrm{d}fCa}{\mathrm{d}t} = \begin{cases} 0, \text{ if } \left(fCa_{inf} > fCa\right) \text{ and } (V > -60) \\ d_{fCa}, \text{ otherwise} \end{cases} \tag{S31}$$

I_{CaL}, *f* gate [5]

$$f_{inf} = \frac{1}{1+e^{\frac{V+20}{7}}} \tag{S32}$$

$$\tau_f = \begin{cases} 100 & ,Early \\ \left(1125 \cdot e^{\frac{-(V+27)^2}{240}} + 80 + \frac{165}{1+e^{\frac{25-V}{10}}}\right) \cdot \begin{cases} \left(1 + 1433 \cdot (Ca_i - 50 \times 10^{-6})\right) & ,f_{inf} > f \\ 1 & ,otherwise \end{cases} & ,Late \end{cases} \tag{S33}$$

$$\frac{\mathrm{d}f}{\mathrm{d}time} = \frac{f_{inf} - f}{\tau_f} \tag{S34}$$

$T - typeCa^{2+}$ Current, I_{CaT} [6]

$$I_{CaT} = g_{CaT} \cdot dCaT \cdot fCaT \cdot (V - E_{Ca}) \tag{S35}$$

I_{CaT}, *dCaT* gate

$$dCaT_{inf} = \frac{1}{1+e^{-\frac{V+26.3}{6}}} \tag{S36}$$

$$\tau_{dCaT} = \frac{1}{1.068 \cdot e^{\frac{V+26.3}{30}} + 1.068 \cdot e^{-\frac{V+26.3}{30}}} \tag{S37}$$

$$\frac{ddCaT}{dtime} = \frac{dCaT_{inf} - dCaT}{\tau_{dCaT}} \tag{S38}$$

I_{CaT}, *fCaT* gate

$$fCaT_{inf} = \frac{1}{1+e^{\frac{V+61.7}{5.6}}} \tag{S39}$$

$$\tau_{fCaT} = \frac{1}{0.0153 \cdot e^{-\frac{V+61.7}{83.3}} + 0.015 \cdot e^{\frac{V+61.7}{15.38}}} \tag{S40}$$

$$\frac{dfCaT}{dtime} = \frac{fCaT_{inf} - fCaT}{\tau_{fCaT}} \tag{S41}$$

Transient outward current, I_{to}

$$I_{to} = g_{to} \cdot r \cdot s \cdot (V - E_K) \tag{S42}$$

I_{to}, *r* gate

$$r_{inf} = \frac{1}{1+e^{\frac{-5-V}{1.8}}} \tag{S43}$$

$$\tau_r = 9.5 \cdot e^{\frac{-(V+40)^2}{1800}} + 0.8 \tag{S44}$$

$$\frac{\mathrm{d}r}{\mathrm{d}time} = \frac{r_{inf} - r}{\tau_r} \tag{S45}$$

I_{to}, *s* gate

$$s_{inf} = \frac{1}{1+e^{\frac{V+20}{5}}} \tag{S46}$$

$$\tau_s = 85 \cdot e^{\frac{-(V+45)^2}{320}} + \frac{5}{1+e^{\frac{V-20}{5}}} + 3 \tag{S47}$$

$$\frac{\mathrm{d}s}{\mathrm{d}time} = \frac{s_{inf} - s}{\tau_s} \tag{S48}$$

Rapid delayed rectifier K^+ current, I_{Kr}

$$I_{Kr} = g_{Kr} \cdot \sqrt{\frac{K_o}{5.4}} \cdot Xr1 \cdot Xr2 \cdot (V - E_K) \tag{S49}$$

I_{Kr}, *Xr1* gate [5]

$$V_{1/2} = -\frac{R \cdot T}{F \cdot Q} \cdot ln\left(\frac{\left(1+\frac{Ca_o}{2.6\times10^{-3}}\right)^4}{L_0 \cdot \left(1+\frac{Ca_o}{0.58\times10^{-3}}\right)^4}\right) - 26 \tag{S50}$$

$$xr1_{inf} = \frac{1}{1+e^{\frac{V_{1/2}-V}{7}}} \tag{S51}$$

$$\alpha_{xr1} = \frac{450}{1+e^{\frac{-45-V}{10}}} \tag{S52}$$

$$\beta_{xr1} = \frac{6}{1+e^{\frac{V+30}{11.5}}} \tag{S53}$$

$$\tau_{xr1} = \alpha_{xr1} \cdot \beta_{xr1} \tag{S54}$$

$$\frac{\mathrm{d}Xr1}{\mathrm{d}time} = \frac{xr1_{inf} - Xr1}{\tau_{xr1}} \tag{S55}$$

I_{Kr}, *Xr2* gate

$$xr2_{inf} = \frac{1}{1+e^{\frac{V+88}{24}}} \quad (S56)$$
$$\alpha_{xr2} = \frac{3}{1+e^{\frac{-60-V}{20}}} \quad (S57)$$
$$\beta_{xr2} = \frac{1.12}{1+e^{\frac{V-60}{20}}} \quad (S58)$$
$$\tau_{xr2} = \alpha_{xr2} \cdot \beta_{xr2} \quad (S59)$$
$$\frac{\mathrm{d}Xr2}{\mathrm{d}time} = \frac{xr2_{inf} - Xr2}{\tau_{xr2}} \quad (S60)$$

Slow delayed rectifier K^+ current, I_{Ks} [5]

$$I_{Ks} = g_{Ks} \cdot Xs^2 \cdot \left(1 + \frac{0.6}{1+\left(\frac{3.8\times10^{-5}}{Ca_i}\right)^{1.4}}\right) \cdot (V - E_{Ks}) \quad (S61)$$

I_{Ks}, *Xs* gate

$$xs_{inf} = \frac{1}{1+e^{\frac{-5-V}{14}}} \quad (S62)$$
$$\alpha_{xs} = \frac{1100}{\sqrt{1+e^{\frac{-10-V}{6}}}} \quad (S63)$$
$$\beta_{xs} = \frac{1}{1+e^{\frac{V-60}{20}}} \quad (S64)$$
$$\tau_{xs} = \alpha_{xs} \cdot \beta_{xs} \quad (S65)$$
$$\frac{\mathrm{d}Xs}{\mathrm{d}time} = \frac{xs_{inf} - Xs}{\tau_{xs}} \quad (S66)$$

Inward rectifier K^+ current, I_{K1}

$$\alpha_{K1} = \frac{0.1}{1+e^{0.06\cdot(V-15-E_K-200)}} \quad (S67)$$
$$\beta_{K1} = \frac{3\cdot e^{0.0002\cdot(V-15-E_K+100)} + e^{0.1\cdot(V-15-E_K-10)}}{1+e^{-0.5\cdot(V-15-E_K)}} \quad (S68)$$
$$xK1_{inf} = \frac{\alpha_{K1}}{\alpha_{K1}+\beta_{K1}} \quad (S69)$$
$$I_{K1} = g_{K1} \cdot xK1_{inf} \cdot \sqrt{\frac{K_o}{5.4}} \cdot (V - E_K) \quad (S70)$$

Hyperpolarization activated funny current, I_f

$$I_f = g_f \cdot Xf \cdot (V - E_f) \quad (S71)$$

I_f, *Xf* gate

$$xf_{inf} = \frac{1}{1+e^{\frac{102.4+V}{7.6}}} \quad (S72)$$
$$\frac{dXf}{dtime} = \frac{xf_{inf} - Xf}{\tau_f} \quad (S73)$$

Na^+/K^+ pump current, I_{NaK}

$$I_{NaK} = \frac{\frac{\frac{P_{NaK}\cdot K_o}{K_o+K_{mk}}\cdot Na_i}{Na_i+K_{mNa}}}{1+0.1245\cdot e^{\frac{-0.1\cdot V\cdot F}{R\cdot T}} + 0.0353\cdot e^{\frac{-V\cdot F}{R\cdot T}}} \quad (S74)$$

Na^{+}/Ca^{2+} exchanger current, I_{NaCa}

$$I_{NaCa} = \frac{K_{NaCa}\cdot\left(e^{\frac{\gamma\cdot V\cdot F}{R\cdot T}}\cdot {Na_i}^3\cdot Ca_o - e^{\frac{(\gamma-1)\cdot V\cdot F}{R\cdot T}}\cdot {Na_o}^3\cdot Ca_i\cdot\alpha\right)}{({Km_{Nai}}^3+{Na_o}^3)\cdot(Km_{Ca}+Ca_o)\cdot\left(1+K_{sat}\cdot e^{\frac{(\gamma-1)\cdot V\cdot F}{R\cdot T}}\right)} \quad \text{(S75)}$$

Ca^{2+} dynamics

$$I_{rel} = \left(\frac{a_{rel}\cdot {Ca_{SR}}^2}{{b_{rel}}^2+{Ca_{SR}}^2} + c_{rel}\right)\cdot d\cdot g \quad \text{(S76)}$$

$$I_{up} = \frac{Vmax_{up}}{1+\frac{{K_{up}}^2}{{Ca_i}^2}} \quad \text{(S77)}$$

$$I_{leak} = V_{leak}\cdot(Ca_{SR}-Ca_i) \quad \text{(S78)}$$

$$g_{inf} = \begin{cases} \frac{1}{1+\left(\frac{Ca_i}{0.00035}\right)^6}, \text{ if } Ca_i \le 0.00035 \\ \frac{1}{1+\left(\frac{Ca_i}{0.00035}\right)^{16}}, \text{ otherwise} \end{cases} \quad \text{(S79)}$$

$$d_g = \frac{g_{inf}-g}{\tau_g} \quad \text{(S80)}$$

$$\frac{\mathrm{d}g}{\mathrm{d}time} = \begin{cases} 0, \text{ if } (g_{inf} > g) \text{ and } (V > -60) \\ d_g, \text{ otherwise} \end{cases} \quad \text{(S81)}$$

$$Ca_{i_{bufc}} = \frac{1}{1+\frac{Buf_c\cdot K_{buf_c}}{\left(Ca_i+K_{buf_c}\right)^2}} \quad \text{(S82)}$$

$$Ca_{sr_{bufsr}} = \frac{1}{1+\frac{Buf_{sr}\cdot K_{buf_{sr}}}{\left(Ca_{SR}+K_{buf_{sr}}\right)^2}} \quad \text{(S83)}$$

$$\frac{\mathrm{d}Ca_i}{\mathrm{d}time} = Ca_{i_{bufc}}\cdot\left(I_{leak}-I_{up}+I_{rel}-\frac{\left(I_{CaL}+I_{CaT}+I_{b_{Ca}}+I_{p_{Ca}}-2\cdot I_{NaCa}\right)}{2\cdot V_c\cdot F}\cdot Cm\right) \quad \text{(S84)}$$

$$\frac{\mathrm{d}Ca_{SR}}{\mathrm{d}time} = \frac{Ca_{sr_{bufsr}}\cdot V_c}{V_{sr}}\cdot(I_{up}-(I_{rel}+I_{leak})) \quad \text{(S85)}$$

Ca^{2+} pump current, I_{pCa}

$$I_{p_{Ca}} = \frac{g_{pCa}\cdot Ca_i}{Ca_i+K_{pCa}} \quad \text{(S86)}$$

Ca^{2+} background current, I_{bCa}

$$I_{b_{Ca}} = g_{bca}\cdot(V-E_{Ca}) \quad \text{(S87)}$$

Reversal potentials

$$E_{Na} = \frac{R\cdot T}{F}\cdot\ln\frac{Na_o}{Na_i} \quad \text{(S88)}$$

$$E_K = \frac{R\cdot T}{F}\cdot\ln\frac{K_o}{K_i} \quad \text{(S89)}$$

$$E_{Ks} = \frac{R\cdot T}{F}\cdot\ln\frac{K_o+P_{kna}\cdot Na_o}{K_i+P_{kna}\cdot Na_i} \quad \text{(S90)}$$

$$E_{Ca} = \frac{0.5\cdot R\cdot T}{F}\cdot\ln\frac{Ca_o}{Ca_i} \quad \text{(S91)}$$

Interaction with in silico fibroblasts

AP of hESC-CM coupled to N_f fibroblasts evolves according to:

$$\frac{dV}{dt} = -\frac{C_m \cdot I_{ion} + N_f \cdot I_{gap}}{C_m} \quad \text{(S92)}$$

where I_{ion} represents the global current density across the hESC-CM membrane and I_{gap} the current flowing through the gap junction.

$$I_{gap} = G_{gap} \cdot (V - V_{fibro}) \quad \text{(S93)}$$

V_{fibro}: fibroblast potential;
$G_{gap} = 1(nS)$: conductance of the hESC-CM - fibroblast coupling;
N_f: number of coupled fibroblasts;

The fibroblast AP evolves according to

$$\frac{dV_{fibro}}{dt} = -\frac{C_{fibro} \cdot I_{fibro}(V_{fibro}) - I_{gap}}{C_{fibro}} \quad \text{(S94)}$$

$C_{fibro} = 1.6(pF)$: fibroblast capacity;
$I_{fibro}(V_{fibro})$: fibroblast global transmembrane current density;

In particular, $I_{fibro}(V_{fibro})$ is the sum of the four currents identified by MacCannell et al. (delayed rectifying K^+ current, inward rectifying K^+ current, Na^+/K^+ pump current, and background Na^+ current) [7]. The background Na^+ current conductance G_{bNa} changed from 0.0095 nS/pF to 0.003 nS/pF. We set this parameter in order to equal the integrated Na^+ influx through the leak pathway to the Na^+ efflux through the Na^+/K^+ pump. We assume this discrepancy to be caused by differences in the cardiomyocyte model.

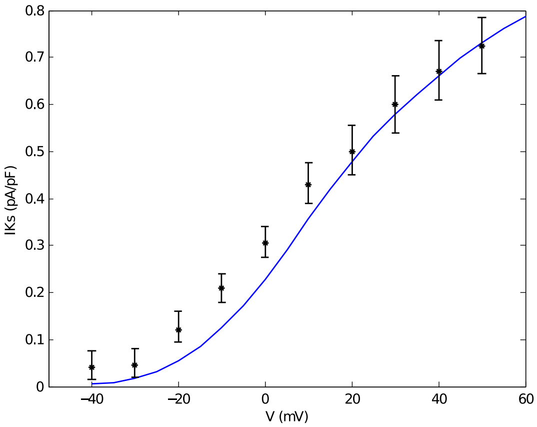

Figure S1. I/V curve for I_{Ks}, obtained with the following protocol: 2 second-test pulses from -40 to 60 mV from an holding potential of -40 mV [8].

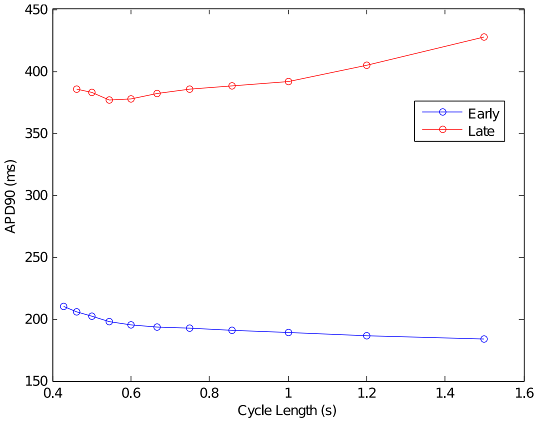

Figure S2. APD90 rate dependence. The used protocol consisted in 300 s with amplitude 0 pA, thus leaving the model reaching spontaneously its own steady state, and then 300 s of stimulation at constant Cycle Length and amplitude 300 pA; then APD90 was computed. At the Early stage no significant APD90 dependence to the stimulus was noticed, while at the Late stage a physiological dependence starts to appear for Cycle Lengths greater than 0.6 s.

References

1. Sartiani L, Cerbai E, Lonardo G, DePaoli P, Tattoli M, Cagiano R, Carratù MR, Cuomo V, Mugelli A: **Prenatal exposure to carbon monoxide affects postnatal cellular electrophysiological maturation of the rat heart: a potential substrate for arrhythmogenesis in infancy.** *Circulation* 2004, **109**(3):419–423.

2. Sartiani L, Bettiol E, Stillitano F, Mugelli A, Cerbai E, Jaconi ME: **Developmental changes in cardiomyocytes differentiated from human embryonic stem cells: a molecular and electrophysiological approach**. *Stem Cells* 2007, **25**(5):1136–1144.

3. Barbieri M, Varani K, Cerbai E, Guerra L, Li Q, Borea PA, Mugelli A: **Electrophysiological basis for the enhanced cardiac arrhythmogenic effect of isoprenaline in aged spontaneously hypertensive rats**. *J Mol Cell Cardiol* 1994, **26**(7):849–860.

4. ten Tusscher KHWJ, Noble D, Noble PJ, Panfilov AV: **A model for human ventricular tissue**. *Am J Physiol Heart Circ Physiol* 2004, **286**(4):H1573–1589.

5. Grandi E, Pasqualini FS, Pes C, Corsi C, Zaza A, Severi S: **Theoretical investigation of action potential duration dependence on extracellular Ca2+ in human cardiomyocytes**. *J Mol Cell Cardiol* 2009, **46**(3):332–342.

6. Maltsev Va, Lakatta EG: **Synergism of coupled subsarcolemmal Ca2+ clocks and sarcolemmal voltage clocks confers robust and flexible pacemaker function in a novel pacemaker cell model.** *Am J Physiol Heart Circ Physiol* 2009, **296**(3):H594–615.

7. MacCannell KA, Bazzazi H, Chilton L, Shibukawa Y, Clark RB, Giles WR: **A mathematical model of electrotonic interactions between ventricular myocytes and fibroblasts.** *Biophys J* 2007, **92**(11):4121–4132.

8. Wang K, Terrenoire C, Sampson KJ, Iyer V, Osteen JD, Lu J, Keller G, Kotton DN, Kass RS: **Biophysical properties of slow potassium channels in human embryonic stem cell derived cardiomyocytes implicate subunit stoichiometry**. *J Physiol (Lond)* 2011, **589**(Pt 24):6093–104.
